# Supplementary material for: Audiovisual and lexical cues do not additively enhance perceptual adaptation
Source: Psychon Bull Rev. 2020 Apr 21;27(4):707–15. doi: 10.3758/s13423-020-01728-5 (PMC7398951; doi:10.3758/s13423-020-01728-5)
Supplement: Supplementary file 1 — (DOCX 89 kb) [file 13423_2020_1728_MOESM1_ESM.docx]

Audiovisual and lexical cues do not additively enhance perceptual adaptation

Shruti Ullas, Elia Formisano,

Department of Cognitive Neuroscience, Faculty of Psychology and Neuroscience,

Maastricht University, 6200 MD Maastricht, The Netherlands

Frank Eisner,

Donders Centre for Cognition, Radboud University Nijmegen,

6500 AH Nijmegen, The Netherlands

& Anne Cutler

MARCS Institute and ARC Centre of Excellence for the Dynamics of Language,
Western Sydney University, Penrith, NSW 2751, Australia

**Corresponding author**:

Shruti Ullas

Department of Cognitive Neuroscience, Faculty of Psychology and Neuroscience,

Maastricht University, 6200 MD Maastricht, The Netherlands

shruti.ullas@maastrichtuniversity.nl, +310433884012

Word count: 2964

**Acknowledgments:** This project was funded by Language in Interaction, within the Netherlands Organization for Scientific Research (NWO) Gravitation program.

**Abstract**

When listeners experience difficulty in understanding a speaker, lexical and audiovisual (or lip-reading) information can be a helpful source of guidance. These two types of information embedded in speech can also guide perceptual adjustment, also known as recalibration or perceptual retuning. With retuning or recalibration, listeners can use these contextual cues to temporarily or permanently reconfigure internal representations of phoneme categories to adjust to and understand novel interlocutors more easily. These two types of perceptual learning, previously investigated in large part separately, are highly similar in allowing listeners to use speech-external information to make phoneme boundary adjustments. This study explored whether the two sources may work in conjunction to induce adaptation, thus emulating real life, in which listeners are indeed likely to encounter both types of cue together. Listeners who received combined audiovisual and lexical cues showed perceptual learning effects similar to listeners who only received audiovisual cues, while listeners who received only lexical cues showed weaker effects compared to the two other groups. The combination of cues did not lead to additive retuning or recalibration effects, however, suggesting that lexical and audiovisual cues operate differently with regard to how listeners utilize them for reshaping perceptual categories. Reaction times did not significantly differ across the three conditions, so none of the forms of adjustment were either aided or hindered by processing time differences. Mechanisms underlying these forms of perceptual learning may diverge in numerous ways despite similarities in experimental applications.

Keywords: recalibration, perceptual retuning, lip-reading, lexical, audiovisual

**Introduction**

Contextual information can impact what listeners perceive they are hearing, and can be helpful when, due to unfamiliar accents, background noise, or idiosyncratic pronunciations, speech is unclear. To adapt to such situations, listeners can draw on cues outside the speech signal, such as lip-reading information or lexical knowledge. The lexical Ganong effect, in which *?esk*, with an ambiguous /d/-/t/ blend replacing /d/, is often heard as *desk* (Ganong, 1980) shows how listeners’ perception of an ambiguous phoneme is influenced by the word in which it occurs. Similarly, in the McGurk effect (where audio of /ba/ accompanying a speaker pronouncing /ga/ prompts a combined percept of /da/; McGurk & MacDonald, 1976), lip-reading information determines what listeners believe they are hearing.

Not only can lexical and audiovisual cues influence the perception of individual speech tokens, but each cue type can reconfigure the listener’s perceptual system. Thus, listeners who heard words such as *giraffe* where an /f/-/s/ blend replaced the /f/ were then more likely to report this blend and similar sounds along a /f/-/s/ continuum as /f/ (Norris, McQueen, & Cutler, 2003). Likewise, listeners who viewed stimuli of a speaker pronouncing /aba/ paired with an auditory /aba/-/ada/ blend then reported hearing /aba/ even when given the ambiguous blend without visual context (Bertelson, Vroomen, & De Gelder, 2003). This audiovisual effect has been termed “recalibration” of phoneme decisions; it can be a conscious action by the listener, and indeed is even taught as a listening strategy (e.g., for taking dictation in second languages). In contrast, the lexical effect, of which listeners are typically unaware, has been referred to as “retuning” to interlocutor-specific articulation. We will here retain this distinction when referring to the two types of adjustment.

McGurk-style fusion percepts between auditory /b/ and visual /g/ (perceived together as /d/) can also result in similar shifts of the perceived boundary along a VOT continuum compared to isolated auditory stimuli without visual accompaniment (Green & Kuhl, 1989). The boundary shift determined by exposure to these fusion percepts can also vary depending on the phoneme pairs tested, such as in a /b/-/p/ pair compared to a /g/-/k/, even though both pairs also vary along the same VOT dimension (Brancazio, Miller, & Paré, 2003). Visual representations of phonetic categories can also undergo shifts guided by lexical information (van der Zande, Jesse, & Cutler, 2013)**.**

Perceptual recalibration and retuning have been extensively studied using lexical and lip-reading cues, but separately, and often with slightly differing experimental designs. Audiovisual recalibration can take place after exposure to as few as eight biasing stimuli (Vroomen, van Linden, de Gelder, & Bertelson, 2007). In contrast, lexically-driven retuning studies have typically used longer exposure phases with around 20 critical items, often embedded into a lexical decision task containing other filler words (see Cutler, Eisner, McQueen, & Norris, 2010, for a review), although Kraljic & Samuel (2007) showed that as few as 10 critical items can also induce lexical retuning. While audiovisual information can induce strong recalibration effects in a short period of time, the effects can dissipate quickly, with increasing numbers of categorization test items (Vroomen et al., 2004)**.** However, lexical retuning appears robust and longer-lasting, measureable up to 24 hours later, again in designs with long exposure phases and usually by inducing a bias towards one particular phoneme (Eisner & McQueen, 2005, 2006; Kraljic & Samuel 2009). The two cue types may therefore operate on different timescales and thus require differing amounts of exposure (Eisner & McQueen, 2006; Vroomen, et al., 2007). Van Linden and Vroomen (2007) directly compared the two processes with matched designs but separate sessions for each cue type; audiovisual cues produced slightly larger effects than lexical cues.

Related research on audiovisual speech processing (see Massaro & Jesse, 2007; Rosenblum, 2010; for overviews) has established that lip-reading information can enhance speech comprehension, especially when the available auditory signal is unclear (Macleod & Summerfield, 1987; Sumby & Pollack, 1954). Lip-reading cues can also enhance the perception of certain types of phonetic information, such as the place of articulation, particularly for bilabial consonants, and can even be available to the listener prior to the onset of auditory phoneme cues (Massaro & Cohen, 1993). Such visual cues however affect reported perception more if a word results (e.g., auditory *besk* with visually presented *desk)*, in contrast to auditory *desk*, visual *besk* where the visual choice makes a non-word (Brancazio, 2004). It has been shown that visual cues can also enhance phoneme perception if visual information is available before auditory signal onset (Mitterer & Reinisch, 2016); but listeners performing a simultaneous interpretation task received no benefit from the presence of lip-reading cues when the auditory signal was clear and free of noise (Jesse, Vrignaud, Cohen, & Massaro, 2000).

Despite this substantial evidence of audiovisual effects on speech perception, prior research has not investigated the perceptual learning effects resulting from combined audiovisual and lexical cues. It remains unknown whether combined cues can induce effects larger than those elicited by either cue on its own. Redundant audiovisual and lexical cues, as listeners are most likely to encounter in real-life, could be more informative and could potentially lead to stronger adaptation effects than either cue in isolation. It may be beneficial for listeners to utilize as many available cues as possible when speech is unclear in order to interpret the ambiguous signal with ease, and thereby shift the underlying categories, rather than to rely on one source of information. However, visual cues may not significantly enhance perceptual learning if the auditory cues alone are sufficiently informative to the listener, or because the necessary exposure for a cue type has not been achieved. By mapping how these cues influence perceptual learning, we hope to enable the extension of current theories of speech perception to account for the role of such information in the process of speech comprehension and speaker adaptation. Although Massaro and Cohen (1993) and Rosenblum (2008) have argued that integrating acoustic and non-acoustic information is crucial for speech comprehension, accounts of speech perception have largely overlooked the contributions of non-acoustic information, especially with regard to perceptual learning (see Weber & Scharenborg, 2012 for a review).

The present study provides the first examination of phoneme boundary retuning given combined lexical and audiovisual information. If multiple sources of biasing information can be additive, we would expect to observe enhanced perceptual learning effects. However, if these cue types differ in the optimal conditions needed (i.e. differences in the amount of exposure needed for effects to be induced) or if one of the two cues can already induce ceiling-level results, then the combination may produce no benefit. To test this, three participant groups were exposed to blocks of either lexical, audiovisual, or combined stimuli containing an ambiguous final phoneme, and in following test phases, ambiguous tokens were presented in a forced-choice categorization task.

**Methods**

**Participants**

Sixty participants were recruited from Maastricht University (32 female; mean age = 23, SD = 2.5 years). All were native Dutch speakers with normal hearing, normal or corrected-to-normal vision, and were compensated monetarily or with study credits. Participants were assigned to one of the three possible conditions (audiovisual, lexical, or combined) randomly, with 20 participants in each group.

**Stimuli**

Three sets of stimuli were constructed for the experiment. All stimuli were created using digital audio and video recordings of a female native Dutch speaker. A set of 16 real Dutch words and 16 pseudo-words were recorded with both /op/ and /ot/ endings, as well as two isolated recordings of the pseudo-words /soop/ and /soot/. For a full list of stimuli with their pronunciations, see Table 1.

The two syllables /op/ and /ot/ (long vowel plus voiceless stop-consonants) were the basis of a ten-step continuum, containing eight steps between these two endpoints, and were created using the *Praat* speech-editing program (Boersma & van Heuven, 2001) based on prior work by McQueen (1991). Similar procedures have been applied by Mitterer, Scharenborg, & McQueen (2013) and Reinisch & Holt (2014) using the STRAIGHT algorithm by Kawahara, Masuda-Katsuse, & De Cheveigné (1999). The two syllables were equated in duration with a 44kHz sampling frequency and with the original pitch contour replaced with an averaged one. The consonant bursts of the two syllables were scaled to have the same peak amplitude and were blended in 10% increments starting from one endpoint. Vowel durations were equated to 186 ms and morphed together in the same manner as consonants. These morphed syllables were spliced onto the ends of the recordings of the words and pseudo-words, with joins made at the zero-crossing closest to the final 50 ms of the vowel to eliminate any co-articulatory cues.

The lexical stimuli were recordings of 16 Dutch words, with eight typically ending in /op/ and the other eight typically ending in /ot/, and matched in frequency and numbers of syllables. None of the selected words could be words if they ended in the alternative phoneme, and none contained any other occurrences of either target phoneme or, with a single exception, of the phonemes /b/ and /d/ that differ from the morphed phonemes only in voicing.

The pseudo-words generated for the audiovisual stimuli, using WinWordGen (Duyck, Desmet, Verbeke, & Brysbaert, 2004), were matched with the words for numbers of syllables. The audio endings of the pseudo-words replaced by the ambiguous steps from the /op/-/ot/ continuum. Video recordings of the pseudo-words contained only the speaker’s mouth pronouncing the items to emphasize the lip-movements, half of which indicated /op/ ending and the other half /ot/ ending. Videos lasted 1200ms on average and no longer than 1500ms**.** The combined audiovisual-lexical stimuli consisted of the same words as the lexical stimuli, with the addition of the video of the speaker pronouncing the words (still centered around the speaker’s mouth). These stimuli contained both lip-movement and lexical cues, while still containing the ambiguous audio ending. All videos had the original audio replaced with the corresponding audio token containing the ambiguous final phoneme.

**Procedure**

Participants were seated in front of a computer in a quiet testing room with audio presented over earphones set to a comfortable volume, using Presentation software (Neurobehavioral Systems). All participants first underwent a pretest by hearing the 10 continuum sounds ranging from /op/ to /ot/ to determine the sound most ambiguous to them. Stimuli sets that are tailored individually allow for equally ambiguous perception across participants, and are comparable in effect size to a pre-selected single midpoint used for all participants (Bruggeman & Cutler, 2019). Each sound was presented 10 times on average, with endpoint sounds presented six to eight times while sounds towards the center were presented 10 to 12 times, and all sounds were presented in random order. Participants responded with a button press for each sound depending on whether they perceived it as /op/ or /ot/. The most ambiguous sound, perceived as either /op/ or /ot/ for the closest average to 50% of responses, was used to select the particular participant’s stimuli set for the retuning experiment.

Following the pre-test, exposure and test stimuli were presented in alternating blocks, for a total of 32 exposure blocks and 32 test blocks. Exposure blocks contained four unique stimuli, each presented twice, for eight items total. Either audio-only recordings of words, videos of pseudo-words, or videos of words were presented in the lexical, audiovisual, and combined conditions, respectively. For the lexical condition, a gray fixation cross was centered on the screen during the eight audio-only trials. In the audiovisual and combined conditions, eight videos were presented during the exposure block. Each individual exposure block induced a bias towards one particular phoneme, (i.e. towards /op/ by presenting only words ending in /op/ in the lexical condition). The phoneme bias of the exposure block was pseudo-randomly alternated every one or two blocks, with 16 blocks inducing a bias towards /p/ and the other 16 towards /t/**,** in order to enable a within-subject measure of perceptual learning results (rather than two separate groups; i.e. one group receiving ambiguous /p/ and the other receiving ambiguous /t/).

A test block followed every exposure block in all conditions, consisting of a categorization task upon the individually-selected ambiguous token from the /op/-/ot/ continuum, and its immediately preceding and following sounds: one more /p/-sounding, one more /t/-sounding. Each sound was presented twice, for six presentations total. After each sound, participants signaled with a button press what they reported hearing (/p/ or /t).

Exposure and test trials lasted 1600 ms each, while test trials were followed by a 1400 ms gap for response. For test blocks in all conditions, a red fixation cross was presented during the sound presentation, followed then by a green fixation cross prompting the participant’s response. Figure 1 provides an overview of the experimental procedure.


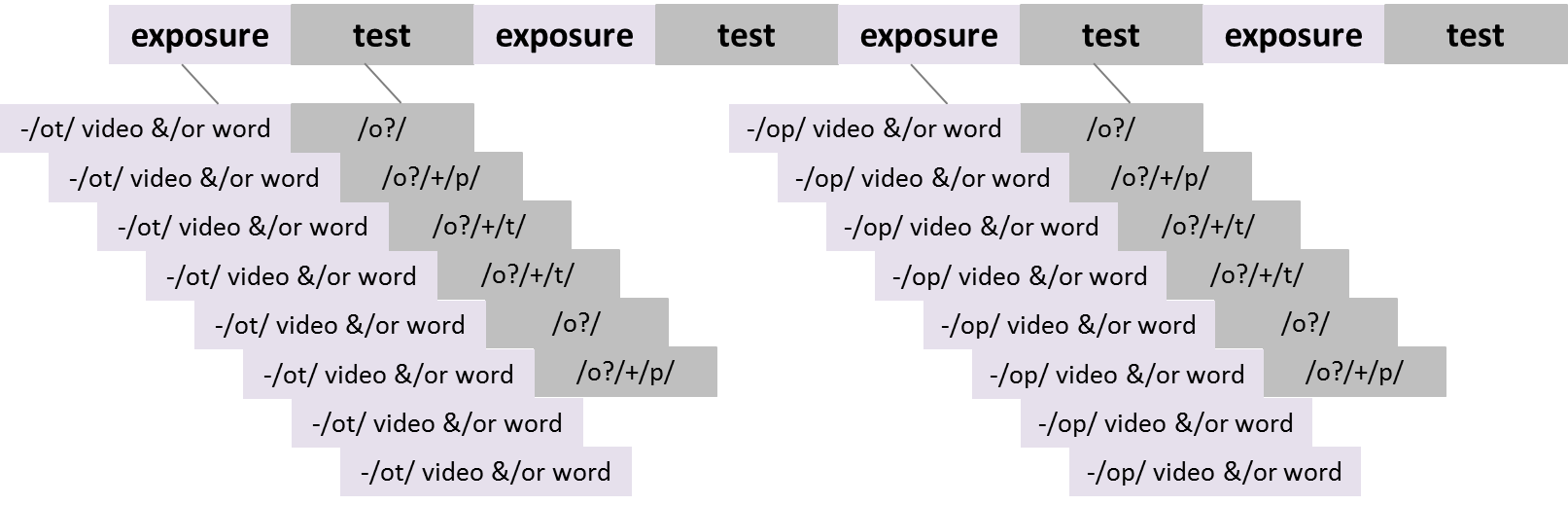


*Figure 1.* Example of blocked exposure-test procedure. In exposure blocks, listeners were presented with eight stimuli (audio recordings of words, videos of pseudo-words, or the combination [videos of words], depending on assigned condition), biased towards /op/ or /ot/ per block. The phoneme bias in each exposure block changed every one or two blocks. In the test blocks following each exposure, listeners heard the most ambiguous sound and its two neighbors (one more /p/-sounding and one more /t/-sounding), and responded whether each sound resembled /op/ or /ot/. The procedure depicted was repeated eight times over the course of the experiment (with pseudo-randomized alternation of phoneme bias in the exposure blocks), such that listeners would be consistently shifting the boundary between the two phoneme endpoints throughout the session.

A separate group of six listeners provided goodness ratings of all of the exposure stimuli (lexical, audiovisual, and combined). Participants were presented with each item three times, and rated them on a scale from 1 to 7, with 1 indicating a clear /p/-ending and 7 indicating a clear /t/-ending (4 if the item was ambiguous). The resulting ratings are shown in Table 2 in the Appendix. These listeners replicated the asymmetry reported by van Linden and Vroomen (2007), where audiovisual stimuli received the highest goodness ratings, followed by the combined stimuli, and with lexical items receiving relatively lower ratings.

**Results**

**Pre-test responses**

Responses during the pre-test were averaged per test sound to determine the most ambiguous token per subject, in order to determine the most appropriate stimulus set. On average, the seventh step was marked as /t/ for 50% of responses and most ambiguous for the majority of participants. Pre-test results are shown in Figure 2. For the individually selected midpoints, the average of /t/ responses for the selected token were 0.41458, 0.44792, and 0.38333, for the audiovisual, lexical, and combined groups respectively.

*Figure 2***.** Pre-test /t/-responses averaged across participants (n = 60) for each sound along the continuum, ranging from clear /ot/ to clear /op/.

**Retuning responses**

Responses during test blocks were entered into a generalized linear mixed model, using the lme4 package in R. *Phoneme bias* during the preceding exposure blocks, *condition* (lexical, audiovisual, or combined), *sound* (the three types of sounds presented during test blocks), and block position (collapsed to range from 1 to 8) were entered into the model as fixed effects. All factors were coded to be centered around zero, except for the test block responses, which were coded as 0 (for /p/) and 1 (for /t/). Within-subjects factors including *phoneme bias*, *sound,* *and block position* in addition to *subjects* were entered as random effects. Random slopes were fitted for within-subjects factors of *phoneme bias*, *sound, and block position,* as well as their interactions. All variables were coded to be centered around zero, but responses were entered as zeroes (/p/) and ones (/t/). The model was created by entering all possible random effects and interactions, while ensuring that the model converged, where all fixed effects correlations were no larger than 0.4. The resulting model was: Response ~ 1 + Phoneme bias * Condition * Sound * Block position + (1 + Phoneme bias * Sound * Block position || Subject; see Table 3).

*
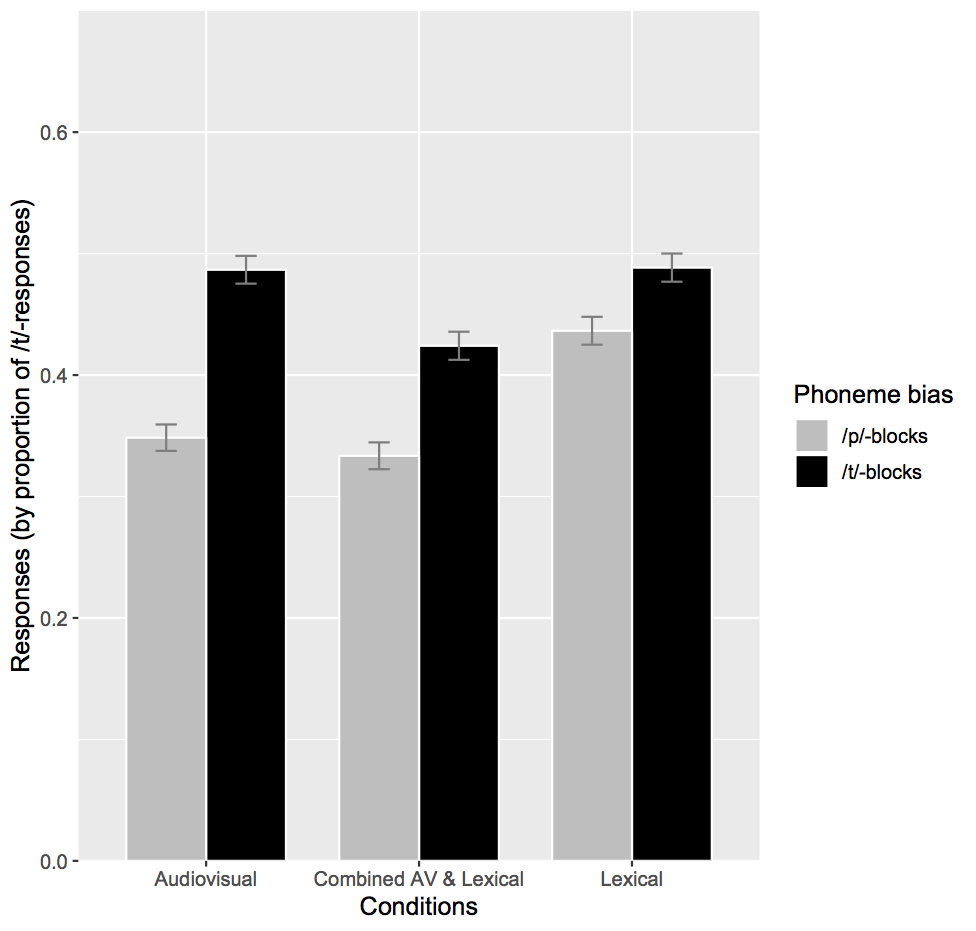
*

*Figure 3*. Recalibration/retuning effects across test sounds for each condition, by proportions of /t/-responses during test blocks, separately by phoneme bias during exposure block.

Effects across the three conditions are depicted in Figure 3. The model showed a significant main effect of *phoneme bias* and the intercept, as well as significant interactions between *phoneme bias* and *condition* and between *phoneme bias* and *block position*. Due to the significant intercept, participants generally had a bias towards responding with /p/ throughout the experiment. However, the main effect of *phoneme bias* indicated that participants responded with significantly more /t/ following /t/-biased exposure, and with /p/ following /p/-biased exposure, demonstrating the retuning/recalibration effect. Due to the interactions between *phoneme bias* and *condition* as well as *phoneme bias* and *block position*, post-hoc t-tests were conducted, and showed that the effect of *phoneme bias* differed between the three conditions and over the series of blocks. On average across the three test sounds, the difference in /t/-responses following /t/- and /p/-biased blocks was larger for the audiovisual and combined conditions (p < 0.0001) while to a lesser extent in the lexical condition (p < 0.01). In addition, the difference in /t/-responses between /t/- and /p/- blocks varied over the block positions, and was significant for all positions in the audiovisual and lexical conditions (p<0.0001), but in the lexical condition, was significant for all blocks (p<0.05) except for the 5^th^ and 7^th^ blocks (p=0.07 and p=0.1316). The subtracted percentage of responses between /t/- and /p/- blocks per block position is shown in Figure 4. The factor *sound* showed no significant main effect or interactions; i.e., the three test sounds did not differ significantly in the proportion of responses elicited.

*Figure 4*. Perceptual learning effects from first to last block. Subtracted percentage of /t/-responses (i.e. /t/-responses after /t/-blocks minus /t/-responses after /p/-blocks) are shown for each block position, separated by the three conditions (audiovisual, lexical, and combined).

**Discussion**

In this study, participants underwent three forms of phoneme boundary adjustments using lexical, audiovisual, or combined stimuli. All three groups successfully showed perceptual learning effects in accordance with the exposure stimuli presented. Audiovisual and combined groups showed stronger effects than the lexical group, but the three groups did not differ significantly from each other. Combined cues resulted in perceptual learning effects similar to audiovisual cues and were numerically larger than lexical retuning effects. An overall bias towards /p/ was observed in all conditions, most likely as a result of the visually noticeable place of articulation of /p/ (bilabial) compared to /t/ (alveolar), as well as the greater lexical information provided by /p/ in word-final positions than /t/. In Dutch, /t/ is often a morphological verb suffix, and does not always carry as useful lexical information in the same manner as /p/. Nevertheless, significant shifts were seen following the phoneme-biased exposure blocks and relative to the pre-test averages to the individually selected ambiguous token as well. From block to block, there was some variation in the amount of perceptual learning effects, particularly as lexical retuning showed some slight reductions in effects (at the fifth and seventh block positions).

Although lexical retuning took place in the study, the observed effects were weaker than those of audiovisual and combined effects**.** The fast, alternating design used in this study may not have provided optimal conditions to elicit such retuning. Previous studies of lexical retuning have often used a single exposure phase, biased only towards one particular phoneme, embedded in a distractor task containing filler words as well (Cutler, et al., 2010). In contrast, in the present study, the phoneme bias was changing throughout the experiment, and was presented in short exposure blocks quickly followed by test blocks. With this design, lexical cues may have insufficient time to build up their potential retuning effects, which are potentially measurable up to 24 hours later in more optimal designs (Eisner & McQueen, 2006). The smaller magnitude of the lexical retuning effect seemed to be driven largely by the lack of /p/-responses after /p/-biased blocks, more so than the /t/-responses after /t/-biased blocks (see Figure 3). The greater proportion of /p/-responses following audiovisual and combined exposure may result from the salience of the visual /p/ more strongly indicating the final /p/ in comparison to the lexical /p/. This finding may also demonstrate the relative rigidity of lexical retuning under the constraints of this study design. Lexical retuning presumably exists for situations involving an unfamiliar pronunciation or accent in which the phoneme bias is in a constant direction. When listeners must continuously update the phoneme category boundary, as in the present study, they may experience difficulty in shifting the boundary in differing directions rather than only in one. Still, lexical retuning can still be accomplished under these restricted conditions of the current study, albeit less robustly.

Audiovisual and combined audiovisual-lexical recalibration were comparable in the obtained effects, and both were larger in comparison to lexically-guided retuning. Notably, combined audiovisual/lexical cues did not result in larger learning effects than audiovisual cues. Although real-life circumstances were more closely emulated by combining lexical and audiovisual cues, which could also allow listeners to readjust faster and more effectively, no such benefit was observed in the pattern of results. It was hypothesized that the compounded cues could have led to an enhanced effect, as listeners had two informative sources available to steer their perceptual adjustments. Instead, the results pointed towards an averaging effect between lexical retuning and audiovisual recalibration. The lexical cues may not have provided any additional benefit to the audiovisual cues during the listeners’ perception of the ambiguous phonemes. If the audiovisual cues alone were enough to induce a perceptual shift in the listeners, then the lexical cues may not have given the listeners any additional support not already available. Audiovisual cues may have therefore produced a ceiling effect, which the addition of lexical cues could not further enhance. Audiovisual integration can also occur at an earlier stage than lexical access (Ostrand et al. 2016), and as the phoneme pair could be distinguished visually by the place of articulation (a bilabial /p/ versus an alveolar /t/) and at an earlier point in time as well, then the subsequent lexical information may not have been able to a further enhance perception. However, relative contributions of visual and lexical information while interpreting ambiguous sounds may also be phoneme-dependent. For example, confusable phonemes sharing the same place of articulation (e.g., /b/, /p/) may be aided more by lexical cues, whereas confusable phonemes that are visually discrepant (e.g., /m/, /n/) may benefit more from lip-reading cues. Thus, adaptation effects may be driven by whichever cues are most salient in a given situation.

Perceptual learning effects per block showed some variation, especially for lexical retuning at the fifth and seventh block positions. As previously mentioned, the design may not be optimal for maximizing lexical retuning, and the variation is a likely consequence. Audiovisual recalibration also showed variation over the blocks, and seemed to decrease from the sixth block towards the end, although not significant statistically. Combined audiovisual-lexical learning appeared more stable over the course of the blocks and less prone to variation. Overall, all perceptual learning effects showed some decreases with prolonged testing, as Vroomen et al. (2004) have previously reported.

Reaction times across the three groups also did not differ significantly (see figure in Appendix). Previously, Brancazio (2004) reported slower responses associated with a visual cue versus an auditory cue for a phoneme within a word, so in the present study we were also interested in whether slower responses would arise with combined audiovisual and lexical effects compared to lexical effects alone. However, Brancazio (2004) did not include phonemes presented without audiovisual or lexical context, whereas in the present study, ambiguous phonemes were presented in test blocks isolated from audiovisual and lexical cues. Our results suggest that Brancazio’s finding reflected a processing time increase to allow for lexical activation; responses in the case of perception of isolated phonemes have no need for such activation, and indeed we found no indication of such reaction time differences.

The combination of ambiguous audio, rather than clear audio, with the audiovisual and lexical cues appears effective in inducing phoneme boundary shifts. One previous study combined both audiovisual and lexical cues in McGurk-style fusion percepts (e.g. auditory *armabillo* paired with visual *armagillo* resulting in a percept of the word *armadillo)* but these stimuli did not induce significant perceptual shifts (Samuel & Lieblich, 2014). McGurk-style fusion stimuli can lead to perceptual shifts (Lüttke, Pérez-Bellido, & de Lange, 2018; Roberts & Summerfield, 1981; Saldaña & Rosenblum, 2005), but such stimuli often combine clear audio of a syllable (/ba/) with an incongruent video of another syllable (such as /ga/), leading to an entirely new percept (/da/). The combination of lexical and audiovisual cues in these McGurk percepts may not allow for perceptual adjustments. In the present study, however, the combination of ambiguous audio with audiovisual and lexical information did prompt a shift in the perceptual boundary. Some relevant acoustic information appears to be necessary to activate lexical and audiovisual representations that allow for recalibration and retuning, even when auditory signals are ambiguous.

Our results show that lexical and audiovisual cues in combination do not jointly enhance perceptual learning. We suggest that the inherent differences in timing between audiovisual and lexical cues is likely to play an important role in how the two cues are integrated to elicit perceptual adjustments. The discrepancy between audiovisual and lexical effects may also be indicative of differences in their underlying structures and networks. Despite the clear similarities between the perceptual learning effects, lexical and audiovisual information seem to diverge in how they operate to adjust phoneme boundaries.

Open practices statement: The data and materials for the experiments reported here are available at (https://hdl.handle.net/10411/UT7PGU) and none of the experiments were preregistered.

References

Bertelson, P., Vroomen, J., & de Gelder, B. (2003). Visual recalibration of auditory speech

identification: a McGurk aftereffect. *Psychological Science*, *14*(6), 592–597.

https://doi.org/10.1046/j.0956-7976.2003.psci_1470.x

Boersma, P., & van Heuven, V. (2001). Speak and unSpeak with PRAAT. *Glot International*,

*5*(9/10), 341–347. https://doi.org/10.1097/AUD.0b013e31821473f7

Brancazio, L. (2004). Lexical influences in audiovisual speech perception. *Journal of*

*Experimental Psychology. Human Perception and Performance, 30*(3), 445-463.

Brancazio, L., Miller, J. L., & Paré, M. A. (2003). Visual influences on the internal structure

of phonetic categories. Perception and Psychophysics, 65(4), 591–601. https://doi.org/10.3758/BF03194585

Bruggeman, L. & Cutler, A. (2019). No L1 privilege in talker adaptation. *Bilingualism,*

*Language and Cognition*. https://doi.org/10.1017/S1366728919000646

Cutler, A., Eisner, F., McQueen, J. M., & Norris, D. (2010). How abstract phonemic

categories are necessary for coping with speaker-related variation. *Laboratory*

*Phonology, 10*, 91–111. https://doi.org/10.1017/CBO9781107415324.004

Duyck, W., Desmet, T., Verbeke, L. P. C., & Brysbaert, M. (2004). WordGen: a tool for word

selection and nonword generation in Dutch, English, German, and French. *Behavior*

*Research Methods, Instruments, and Computers*, *36*(3), 488–499.

https://doi.org/10.3758/BF03195595

Eisner, F., & McQueen, J. M. (2005). The specificity of perceptual learning in speech

processing. *Perception & Psychophysics*, *67*(2), 224–238.

https://doi.org/10.3758/BF03206487

Eisner, F., & McQueen, J. M. (2006). Perceptual learning in speech: stability over time. *The*

*Journal of the Acoustical Society of America, 119*(4), 1950-1953.

https://doi.org/10.1121/1.2178721

Ganong, W. F. (1980). Phonetic categorization in auditory word perception. *Journal of*

*Experimental Psychology. Human Perception and Performance*, *6*(1), 110–125.

https://doi.org/10.1037/0096-1523.6.1.110

Green, K. P., & Kuhl, P. K. (1989). The role of visual information in the processing of place

and manner features in speech perception. *Perception & Psychophysics*, *45*(1), 34–42.

https://doi.org/10.3758/BF03208030

Jesse, A., Vrignaud, N., Cohen, M. M., & Massaro, D. W. (2000). The processing of

information from multiple sources in simultaneous interpreting. *Interpreting*, *5*(2), 95

115. https://doi.org/10.1075/intp.5.2.04jes

Kawahara, H., Masuda-Katsuse, I., & De Cheveigné, A. (1999). Restructuring speech representations using a pitch-adaptive time-frequency smoothing and an instantaneous-frequency-based F0 extraction: Possible role of a repetitive structure in sounds. *Speech Communication*, *27*(3), 187–207. https://doi.org/10.1016/S0167-6393(98)00085-5

Kraljic, T., & Samuel, A. G. (2007). Perceptual adjustments to multiple speakers. *Journal of Memory and Language, 56*(1), 1–15. https://doi.org/10.1016/j.jml.2006.07.010

Kraljic, T., & Samuel, A. G. (2009). Perceptual learning for speech. *Perception &*

*Psychophysics*, *71*(3), 481–489. https://doi.org/10.3758/APP

Lüttke, C. S., Pérez-Bellido, A., & de Lange, F. P. (2018). Rapid recalibration of speech

perception after experiencing the McGurk illusion. *Royal Society Open Science*, *5*(3).

https://doi.org/10.1098/rsos.170909

Macleod, A., & Summerfield, Q. (1987). Quantifying the contribution of vision to speech

perception in noise. *British Journal of Audiology, 21*(2), 131-141.

https://doi.org/10.3109/03005368709077786

Massaro, D. W., & Cohen, M. M. (1993). Perceiving asynchronous bimodal speech in

consonant-vowel and vowel syllables, *Speech Communication, 13*(1-2), 127–134.

Massaro, D. W., & Jesse, A. (2007). Audiovisual speech perception and word recognition.

*The Oxford Handbook of Psycholinguistics,* 19-36.

https://doi.org/10.1093/oxfordhb/9780198568971.013.0002

McGurk, H., & MacDonald, M. (1976). Hearing lips and seeing voices. *Nature, 264*(5588),

746.

McQueen, J. M. (1991). The influence of the lexicon on phonetic categorization: stimulus quality in word-final ambiguity. *Journal of Experimental Psychology: Human Perception and Performance*, *17*(2), 433–443. https://doi.org/10.1037/0096-1523.17.2.433

Mitterer, H., & Reinisch, E. (2016). Visual speech influences speech perception immediately

but not automatically. *Perception & Psychophysics*, *79*(2), 660–678.

https://doi.org/10.3758/s13414-016-1249-6

Mitterer, H., Scharenborg, O., & McQueen, J. M. (2013). Phonological abstraction without phonemes in speech perception. *Cognition*, *129*(2), 356–361. https://doi.org/10.1016/j.cognition.2013.07.011

Norris, D., McQueen, J. M., & Cutler, A. (2003). Perceptual learning in speech. *Cognitive*

*Psychology*, *47*(2), 204–238. https://doi.org/10.1016/S0010-0285(03)00006-9

Ostrand, R., Blumstein, S. E., Ferreira, V. S., & Morgan, J. L. (2016). What you see isn’t

always what you get: auditory word signals trump consciously perceived words in

lexical access. *Cognition*, *151*, 96–107. https://doi.org/10.1016/j.cognition.2016.02.019

Reinisch, E., & Holt, L. L. (2014). Lexically guided phonetic retuning of foreign-accented speech and its generalization. *Journal of Experimental Psychology. Human Perception and Performance*, *40*(2), 539–555. https://doi.org/10.1037/a0034409

Roberts, M., & Summerfield, Q. (1981). Audiovisual presentation demonstrates that selective

adaptation in speech perception is purely auditory. *Perception & Psychophysics*,

*30*(4), 309–314. https://doi.org/10.3758/BF03206144

Rosenblum, L. D. (2008). Speech perception as a multimodal phenomenon. *Current Directions in Psychological Science*, *17*(6), 405–409. https://doi.org/10.1111/j.1467-8721.2008.00615.x

Rosenblum, L. D. (2010) *See what I'm saying: the extraordinary powers of our five senses.*

W. W. Norton & Company, New York, NY.

Saldaña, H. M., & Rosenblum, L. D. (1994). Selective adaptation in speech perception using a

compelling audiovisual adaptor. *The Journal of the Acoustical Society of America,*

*95*(6), 3658-3661. https://doi.org/10.1121/1.409935

Samuel, A. G., & Lieblich, J. (2014). Visual speech acts differently than lexical context in

supporting speech perception. *Journal of Experimental Psychology: Human*

*Perception and Performance*, *30*(9), 1740–1747.

https://doi.org/10.3174/ajnr.A1650.Side

Sumby, W. H., & Pollack, I. (1954). Visual contribution to speech intelligibility in noise. *The*

*Journal of the Acoustical Society of America*. *26*(2), 212-215. https://doi.org/10.1121/1.1907309

Van der Zande, P., Jesse, A., & Cutler, A. (2013). Lexically guided retuning of visual phonetic categories. *The Journal of the Acoustical Society of America*, *134*(1), 562–571. https://doi.org/10.1121/1.4807814

Van Linden, S., & Vroomen, J. (2007). Recalibration of phonetic categories by lipread speech

versus lexical information. *Journal of Experimental Psychology: Human Perception*

*and Performance*, *33*(6), 1483–1494. https://doi.org/10.1037/0096-1523.33.6.1483

Vroomen, J., Van Linden, S., Keetels, M., De Gelder, B., & Bertelson, P. (2004). Selective adaptation and recalibration of auditory speech by lipread information: dissipation. *Speech Communication*, *44*(1-4 SPEC. ISS.), 55–61. https://doi.org/10.1016/j.specom.2004.03.009

Vroomen, J., van Linden, S., de Gelder, B., & Bertelson, P. (2007). Visual recalibration and

selective speech adaptation in auditory-visual speech perception: contrasting build-up

courses. *Neuropsychologia, 45*(3), 572-577.

Weber, A., & Scharenborg, O. (2012). Models of spoken-word recognition. *Wiley Interdisciplinary Reviews: Cognitive Science*, *3*(3), 387–401. https://doi.org/10.1002/wcs.1178

**Table 1: Words & pseudowords**

1. /op/ words:

| Hoop | [hoʊp] |
| --- | --- |
| Siroop | [sɪʀoʊp] |
| Aanloop | [aːnloʊp] |
| Afkoop | [ɑfkoʊp] |
| Wanhoop | [ʋɑnhoʊp] |
| Geweerloop | [ɣəʋeːrloʊp] |
| Horoscoop | [ɦɔʀɔscoʊp] |
| Kussensloop | [kʏsənsloʊp] |

1. /ot/ words:

| Vloot | [vloʊt] |
| --- | --- |
| Afsloot | [ɑfsloʊt] |
| Vennoot | [vɛnoʊt] |
| Vergroot | [vəʀɣʀoʊt] |
| Walnoot | [ʋaːlnoʊt] |
| Hazelnoot | [ɦɑzəlnoʊt] |
| Levensgroot | [lɛvənsɣʀoʊt] |
| Middenmoot | [mɪdənmoʊt] |

1. /op/ pseudo-words:

| Smoop | [smoʊp] |
| --- | --- |
| Aaroop | [aːʀoʊp] |
| Miloop | [mɪloʊp] |
| Onsoop | [ɔnsoʊp] |
| Weloop | [ʋəloʊp] |
| Acenkoop | [ɑsəŋkoʊp] |
| Lakeroop | [lɑkəʀoʊp] |
| Senkenloop | [sɛŋkənloʊp] |

1. /ot/ pseudo-words:

| Vroot | [vʀoʊt] |
| --- | --- |
| Faloot | [fɑloʊt] |
| Geroot | [ɣəʀoʊt] |
| Mevoot | [məvoʊt] |
| Neuloot | [nø:loʊt] |
| Frieseloot | [fʀisəloʊt] |
| Leuveroot | [lø:vəʀoʊt] |
| Sanekoot | [sɑnəkoʊt] |

**Table 2: Stimuli ratings**

Ratings of the stimuli (n=6) on a scale from 1-7 (1 for clear /p/, 7 for clear /t/, 4 for ambiguous).

|  | /p/-ending | /t/-ending |
| --- | --- | --- |
| Lexical (audio words) | 3.29166667 | 4.91666667 |
| Audiovisual (audio+video pseudowords) | 2.36111111 | 5.5625 |
| Combined (audio+video words) | 2.64583333 | 5.40277778 |

**Table 3: Retuning/recalibration results**

Response ~ 1 + Phoneme bias * Condition * Sound * Block position + (1 + Phoneme bias * Sound * Block position || Subject)

|  | Estimate | Std. Error | z value | Pr(>\|z\|) |  |
| --- | --- | --- | --- | --- | --- |
| (Intercept) | -0.38632 | 0.077687 | -4.973 | 6.60E-07 | *** |
| Phoneme | 0.219164 | 0.027841 | 7.872 | 3.49E-15 | *** |
| Condition | 0.098318 | 0.095028 | 1.035 | 0.30085 |  |
| Sound | 0.004709 | 0.034309 | 0.137 | 0.89083 |  |
| Block | 0.021641 | 0.011294 | 1.916 | 0.05534 |  |
| Phoneme*Condition | -0.10528 | 0.033877 | -3.108 | 0.00189 | ** |
| Phoneme*Sound | -0.02038 | 0.037177 | -0.548 | 0.58361 |  |
| Condition*Sound | 0.031938 | 0.041826 | 0.764 | 0.4451 |  |
| Phoneme*Block position | -0.01588 | 0.007372 | -2.154 | 0.03125 | * |
| Condition*Block position | -0.02189 | 0.013761 | -1.591 | 0.1117 |  |
| Sound*Block position | 0.010039 | 0.013084 | 0.767 | 0.44291 |  |
| Phoneme*Condition*Sound | 0.013674 | 0.045333 | 0.302 | 0.76292 |  |
| Phoneme*Condition*Block position | 0.011169 | 0.008955 | 1.247 | 0.21234 |  |
| Phoneme*Sound*Block position | -0.01966 | 0.01462 | -1.345 | 0.17866 |  |
| Condition*Sound*Block position | 0.006478 | 0.015955 | 0.406 | 0.68475 |  |
| Phoneme*Condition*Sound*Block | 0.003955 | 0.017842 | 0.222 | 0.82458 |  |

*Significance: ***p<0.0001; **p<0.01; *p<0.05*


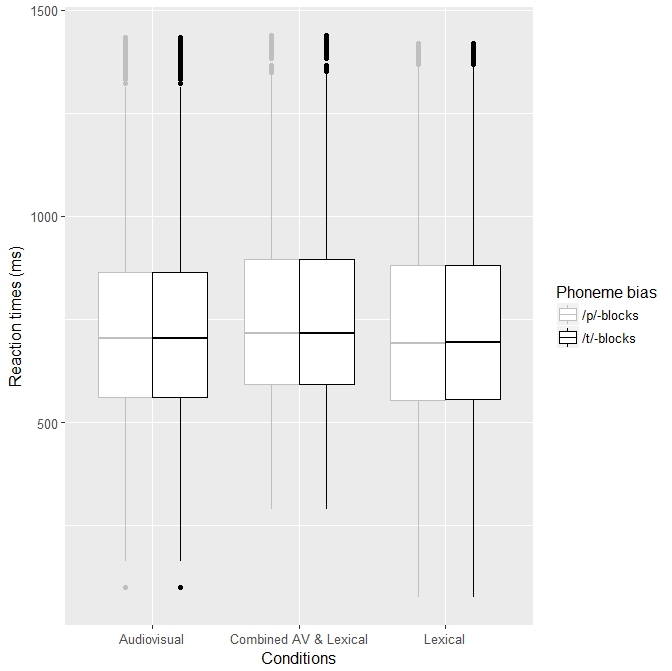


*Supplemental figure:* Reaction times across the three testing groups, separately by phoneme bias during the preceding exposure block.
